# A comprehensive insight of innovations and recent advancements in nanocarriers for nose-to-brain drug targeting

**DOI:** 10.1080/15685551.2025.2464132

**Published:** 2025-02-10

**Authors:** Naeem Mubarak, Muhammad Ahsan Waqar, Asad Majeed Khan, Zainab Asif, Aima Subia Alvi, Aqsa Arshad Virk, Sakeena Amir

**Affiliations:** a Department of Pharmacy Practice, Faculty of Pharmaceutical Sciences, Lahore University of Biological and Applied Sciences, Lahore, Pakistan; b Department of Pharmaceutics, Faculty of Pharmaceutical Sciences, Lahore University of Biological and Applied Sciences, Lahore, Pakistan

**Keywords:** Central nervous system, nose-to-brain, blood-brain barrier, nanotechnology, nanocarriers, polymeric nanoparticles

## Abstract

Central Nervous System (CNS) disorders are the leading cause of illness and affect the everyday lives of people all around the globe and are predicted to increase tremendously in the upcoming decades. Traditional methods of delivering drugs to the CNS face considerable limitations. Nose-to-brain targeting offers a promising alternative that bypasses the blood-brain barrier (BBB), enabling targeted drug administration to the central nervous system (CNS). Nanotechnology has brought forward innovative solutions to the challenges of drug delivery in CNS disorders. Nanocarriers such as liposomes, nanoparticles, nanoemulsions and dendrimers can enhance drug stability, bioavailability, and targeted delivery to the brain. These nanocarriers are designed to overcome physiological barriers and provide controlled and sustained drug release directly to the CNS. Nanocarrier technology has made significant strides in recent years, enabling more effective and targeted delivery of drugs to the brain. With recent advancements, intranasal delivery coupled with nanocarriers seems to be a promising combination that can provide better clinical profiles, pharmacokinetics, and pharmacodynamics for neurodegenerative disorders. This study focuses on exploring the nose-to-brain drug delivery system, emphasizing the use of various nanocarriers designed for this purpose. Additionally, the study encompasses recent advancements in nanocarrier technology tailored specifically to improve the efficiency of drug administration through the nasal route to the brain.

## 1. Introduction

Central nervous system (CNS) diseases are among the primary causes of death and disability as the world’s population ages [1]. According to the World Health Organization (WHO), depression affects over 350 million people globally, with millions committing suicide each year. Parkinson’s disease (PD) and Alzheimer’s disease (AD) stand out as two of the most significant health challenges of our century, both causing severe impairment. These are becoming increasingly common among the ageing population [2]. These diseases typically include gradual degradation and neuronal death, making them widespread and difficult to cure [3]. Neurodegenerative diseases are a collection of ailments defined by the gradual loss of neuronal subtypes [4,5]. Anti-depressant drugs have poor central nervous system distribution across the blood-brain barrier. Therefore, multiple potential techniques for drug administration to the brain have been studied. One technique is to administer the drug via the intranasal route. The intranasal dosage form increases the drug’s bioavailability. Because of its high blood flow, vast surface area, and the ability to avoid the first pass of metabolism, the nasal epithelium allows for fast drug absorption into the brain [6,7]. The intranasal route successfully delivers the drug to the brain without passing through the blood-brain barrier because of the particular link between the nose and the CNS [8,9]. Drugs can be targeted to the brain more successfully by passing through the olfactory area of the nose and absorbing the substance [10]. The brain’s sole interaction with the external environment is the nasal passage. The branching axons of the 12th cranial nerve establish connections between the superior and posterior portions of the nasal cavity. These nerve fibres penetrate through the mucosal membrane and establish a connection with the outer surroundings without relying on a peripheral sensory receptor. These nerves serve as chemoreceptors, detecting food aromas and playing a crucial role in social behavior. Furthermore, these nerves offer a possible route for the immediate delivery of drugs into the CNS [5–7]. Recent research has revealed the possibility of a functional pathway (also known as ‘nose-to-brain’ transport) for drugs. These nerve fibres that connect directly to the CNS, bypassing traditional sensory receptors, offer a unique pathway for drug delivery. This mechanism aligns with the growing interest in nanocarriers, which are being explored for their potential to enhance drug delivery to the brain.

Nanocarriers have an intriguing role in delivering drugs to the central nervous system (CNS); therefore, imaging agents or therapies for CNS diseases rely heavily on them nowadays. Nanocarriers have been found to actively and successfully breach the blood-brain barrier and deeply penetrate damaged brain tissue. Nanocarriers are associated with greater surface area, stability, and responsiveness. CNS nanocarriers possess novel, sophisticated, and adaptive capabilities to carry out diagnostic and therapeutic functions simultaneously; however, additional improvements are necessary to facilitate the wider adoption of this technology in clinical settings.

Nanocarriers manufactured for the management of various diseases are a cutting-edge, promising, and rapidly expanding subject of study [11]. Nanoparticles, among other things, include medicines used to treat neuropsychiatric disorders. All mental diseases need long-term therapy, with patients requiring continual drug for days or even years [12]. As a result, the drug must be given using nanocarriers, which allow for a prolonged release of the drug [13]. Moreover, other types of nanoparticles, including gold, silica, hydrogel, liposomes, and others, are currently under investigation for their potential use in delivering drugs to the central nervous system [14,15]. These nanoparticles can significantly improve their therapeutic advantages by selectively delivering a much greater quantity of drug to diseased areas instead of overloading the entire body with drugs [16]. Furthermore, biocompatibility may be achieved in most nanocarriers by the use of new manufacturing techniques and surface functionalization.

This review provides a comprehensive overview of the nose-to-brain drug delivery system, highlighting its superiority in drug delivery. This study has also explored novel nanocarriers in nose-to-brain drug delivery, discussing their advantages and limitations in detail. Moreover, recent advancements in nanocarrier technology for nose-to-brain delivery have also been discussed along with their future perspectives.

## 2. Drug delivery systems

**Figure 1.**
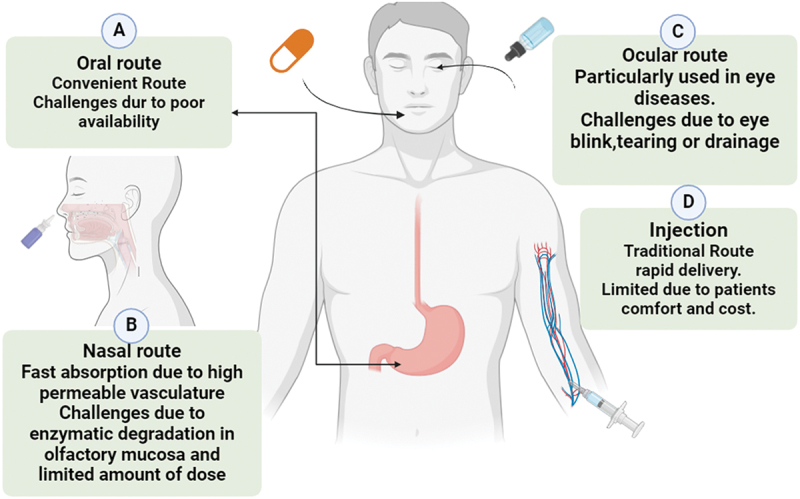
Various routes of administration.

The drug administration route refers to the pathway via which the medicine or dosage form is delivered to the body for the purpose of treating a condition. Several routes for the delivery of drugs as shown in Figure 1 play an important role in the bioaccumulation of the drug in the body [17,18]. It is the pathway through which a pharmaceutical product is delivered to a living organism to achieve the desired therapeutic effect [19,20]. The oral route for the delivery of a drug is the most recommended method for the administration of a drug to get the desired therapeutic effect. In this route, the pharmaceutical dosage forms in the mouth and is engulfed. It can be administered by patients themselves [21,22]. It is very easy to administer and free from pain. However, there is a disadvantage of the oral route, which may cause irritability to the gastric mucosa and cause nausea and vomiting. Transdermal drug delivery systems (TDDS), also known as ‘Transdermal patches’ or ‘Skin patches,’ are adhesive patches that are applied over the skin to transfer drugs into the bloodstream [23]. There are certain factors that affect transdermal permeation, such as skin condition, age, blood supply, metabolism, hydration, and environmental factors such as sunlight, cold season, and air pollution [24]. A drug which is delivered by transdermal route is of great importance as the risk of overdose or underdoes is eliminated and provides easy termination of the drug.

Furthermore, it allows for local and systematic treatment [25–27]. The drug delivery system is poorly understood, but a large variety of drugs have been administered to the lungs through oral inhalation to treat diseases such as COPD and asthma [28,29]. It is an optimal drug delivery system which specifically deposits the drug at a particular pulmonary site; it is not dependent on any pathophysiological parameters; further studies are addressing the unique molecular – biochemical, and physiological characteristics for optimizing the current medication [30,31].

The administration of a substance through the parenteral route involves the operation of a substance on the body in such a way that it passes the Gastrointestinal Tract. Administration of a small amount of drug in the form of a solution is called an injection. At the same time, The administration of drugs in large quantities is called infusion. In both injection and infusion, the drug is administered through the skin by needle [32]. Those pharmacological substances with narrow therapeutic windows and poor bioavailability, which are administered through the parenteral route, are the most efficacious for the administration of drugs, especially for those drugs which are given to insensible patients [33–35]. For the maintenance of therapeutic effective concentration, the drug must be regularly injected, which may cause irritation in the patient [36,37].

Nasal and pulmonary routes impart great importance for treating human disease [38,39]. The intra-nasal administration of drugs offers an interesting alternative to achieving systematic therapeutic outcomes [40]. The nasal route provides the easiest route, which is generally well tolerated and provides a large surface area. Therefore, when the product is under development, some biopharmaceutical considerations need to be focused on, such as what they are intended for localized delivery, systemic delivery, and single or repetitive administration. The nasal route is more advantageous compared to the parental route in terms of convenience and its great potential to deliver it directly to the brain organ (the vital and most functional organ of the body).

Moreover, it is protected by blood-brain barrier [41,42]. Nasal products contain a great variety. Nasal drops and sprays, nasal gels, nasal suspension and emulsion, nasal micelle and liposomal formulation, powders, and microparticles [43,44]. Developing a novel drug delivery system for already available drugs not only enhances drug progression in terms of efficacy and safety but also polishes up patient compliance and therapeutic outcomes to a greater extent.

## 3. Nose to brain drug delivery system

**Figure 2.**
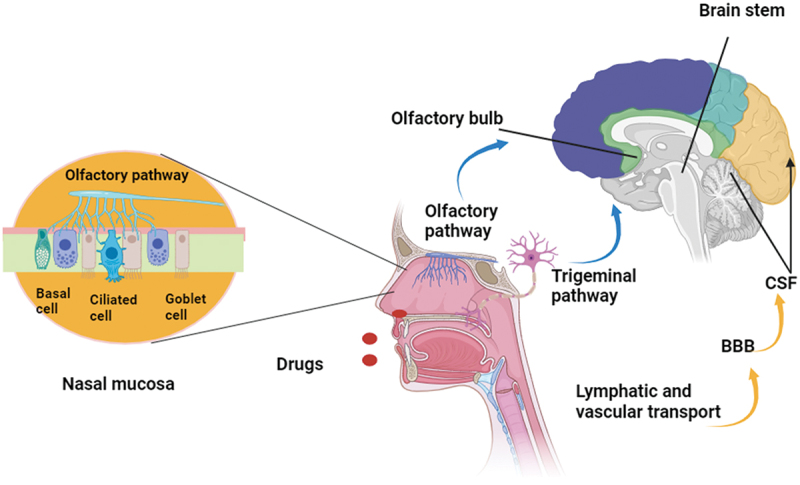
Various pathways of nose to brain delivery.

Nose-to-brain delivery can be achieved through four main routes, which are the olfactory pathway, trigeminal pathway, permeation across nasal mucosa, and lymphatic drainage as depicted in Figure 2 [45]. The olfactory route is the primary pathway for the direct delivery of drugs to the brain through the olfactory epithelium by circumventing the blood-brain barrier [46]. The drugs are transported via the olfactory nerve (via nerve bundles and along axons that cross the cribriform plate), reach the olfactory bulb and then to the deep parts of the brain. This pathway is situated below the cribriform plate of ethmoid bone that separates the nasal cavity from the cranial cavity. The cribriform plate, surrounded by an arachnoid membrane, which contains subarachnoid cerebrospinal fluid between the nerve and membrane, is penetrated by olfactory neurons [47]. This terminates as olfactory sensory endings (OSE), which penetrate through olfactory mucosa. So, the drugs can enter into the brain parenchyma via cerebrospinal fluid [48]. There are three pathways through which transport can occur, i.e., intracellular axonal transport via olfactory nerve following pinocytosis or endocytosis into the olfactory bulb, transcellular transport by endocytosis between sustentacular cells for lipophilic drugs, and paracellular transport across the tight junctions of sustentacular cells [49].

Drugs given via the nose may interact with the nerve terminals of the trigeminal nerve, which is found in the nasal mucosa [50]. A pathway is provided by these neurons for drugs to reach other areas of the brain, including the brainstem and other areas of the central nervous system, bypassing the BBB [51]. The trigeminal nerve transmits sensory information to the brain and spinal cord from the nasal cavity, oral cavity, eyelids, and cornea through the ophthalmic division (V1), maxillary division (V2), or mandibular division (V3) of the nerve [52]. The trigeminal nerve’s ophthalmic division branches innervate the anterior section of the nose and the dorsal nasal mucosa. Maxillary division branches innervate the lateral walls of the nasal mucosa. The mandibular division, which has no direct relationship to the nasal cavity, includes the lower jaw and teeth. All three branches connect at the trigeminal ganglion and extend centrally to the brain, ending in spinal trigeminal nuclei found in the brainstem [53]. While discussing nose-to-brain drug delivery, biopharmaceutical considerations are crucial. Drug size plays a significant role, as larger molecules may face challenges in crossing the nasal mucosa and reaching the brain effectively. Lipophilicity is another key factor, with more lipophilic compounds having better permeation through the nasal membranes, enhancing brain uptake. Additionally, specialized formulations, such as nanoparticles, liposomes, or gel-based systems, are often employed to improve drug stability, enhance mucosal absorption, and facilitate efficient transport across the blood-brain barrier. These formulations ensure that the drug reaches the target sites within the brain at therapeutic concentrations.

**Figure 3.**
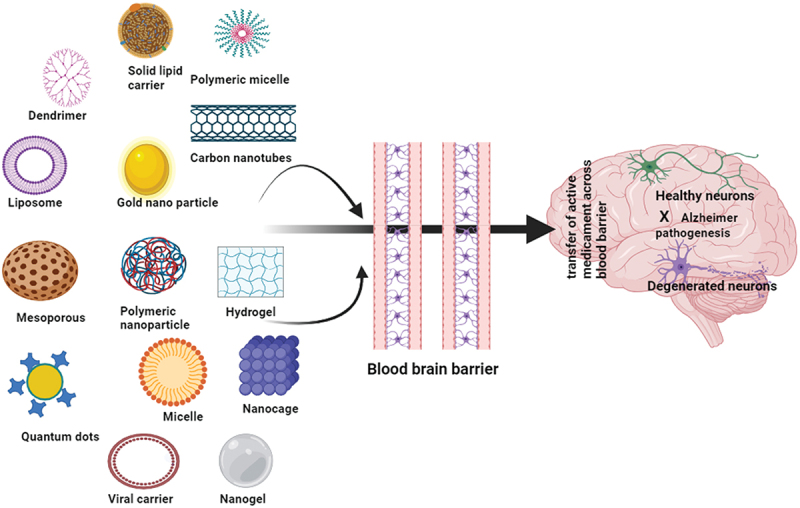
Various approaches for delivery of drug towards brain.

Drugs may also permeate through the nasal mucosa, entering the circulatory system and then reaching the brain via the bloodstream [51]. This pathway may require crossing the BBB, which can restrict the penetration of drugs into the brain, however incorporating the drug into a drug delivery system can overcome this barrier (Figure 3). The lymphatic drainage system maintains overall homeostasis, tissue solute and water balance, immunity and metabolism. It is made up of a network of capillaries, blind-ended that drain themselves into larger vessels and are thus responsible for removing lymph-containing waste and cells from the interstitial fluid surrounding most organs, tissues, proteins, and fluid. In the end, the lymphatic system drains into the venous system for recirculation. Brain parenchyma does not contain conventional lymphatic vessels; however, the central nervous system contains its unique lymphatic drainage structures. Several studies revealed that intranasal administration of drugs may use drainage channels of the lymphatic system, which enables the drugs to bypass the BBB and enter the brain interstitial via the lymphatic system [54]. Various approaches along with their benefits and limitations for targeting the brain via the intranasal route have been mentioned in Table 1.

**Table 1.** Benefits and limitations of approaches for brain targeting via Intranasal Route.

Techniques	Advantages	Limitations	Reference
Gold nanoparticles	Photodynamic and photothermal therapy, x-ray imaging and drug delivery systems	Neurotoxic effects such as seizures, astrogliosis and judgment impairments	[55]
Nanoparticles	Brain targeting, active drug targeting	Cross the BBB	[56]
Silver nanoparticles	Anti-inflammatory, drug delivery systems	Neurotoxicity	[57]
Magnetic nanoparticles	Biosensors, contrast agents for MRI, targeted gene or drug delivery	Limited biocompatibility, cytotoxicity, poor stability, uncontrolled shape, uneven size distribution	[58]
Nanoparticles designed for brain diagnostics or imaging	Better imaging results can cross the BBB under disease conditions by increasing the permeability	Difficulty understanding of the dynamic changes in the BBB	[59–61]
Brain permeability regulators	Temporary BBB opening	Inconsistencies in findings between rats and humans	[62]
Increased uptake of drugs by the use of non-invasive techniques	Decrease the activity of efflux transporters	High toxicity	[55,63]
Viral vectors	Enhanced efficiency in the process of gene transfection	Brain intracerebral injection, safety concerns, fear of high dosage administration	[64–66]
Exosomes	Gene delivery to CNS can cross the BBB	Difficult drug loading, poor pharmacokinetics	[67,68]
Niosomes	Reduced dose, increased bioavailability, stable, targeted drug delivery	Special equipment is required, time-consuming, inefficient drug loading	[69–72]
Drug distribution via active transporters in the blood-brain barrier (BBB)	Penetrate the blood-brain barrier by intravenous injection.	Beneficial for administering compact drug substances	[73,74]
Drug delivery in the diseased state across the permeable BBB	Easily penetrate the BBB	Transformations in BBB are not completely understood	[62,63,75]
Use of alternate administration route	Administer a substance nasally to circumvent the blood-brain barrier.	Intended for use with minimal dosage only	[76,77]

### 3.1. Blood-brain barrier

The blood-brain barrier is a barrier that restricts the entry of toxins and some other compounds into the brain via blood and protects the brain’s neural tissues [78]. The paracellular tight junction and the intercellular adherents junction are its two constituent cellular junctions [79]. Paracellular tight junctions play a key role in the permeability of the blood-brain barrier (BBB). In adults, the BBB is present in the form of a complex cellular network. The main components are highly specialized basal membrane, brain endothelial cells, plenty of pericytes rooted in the basal membrane, and astrocytic end-feet [78]. BBB allows the vessels to regulate the movement of ions and molecules. CNS homeostasis allows neuronal function and protection from toxins in tissues of the brain. Understanding that all these cells interact with brain barrier characteristics is also important to understanding how our brain works in a diseased body [80]. The blood-brain barrier has a role in the influx and efflux of biological materials, which are important for the metabolism and function of the brain. The BBB maintains the homeostatic process of the brain [81]. It has a very complex and dynamic structure. It consists of endothelial cells, pericytes, and astrocytes. There are two components: one is cellular, and the other is non-cellular. The cellular component is of higher importance than Its non-cellular component [82].

**Figure 4.**
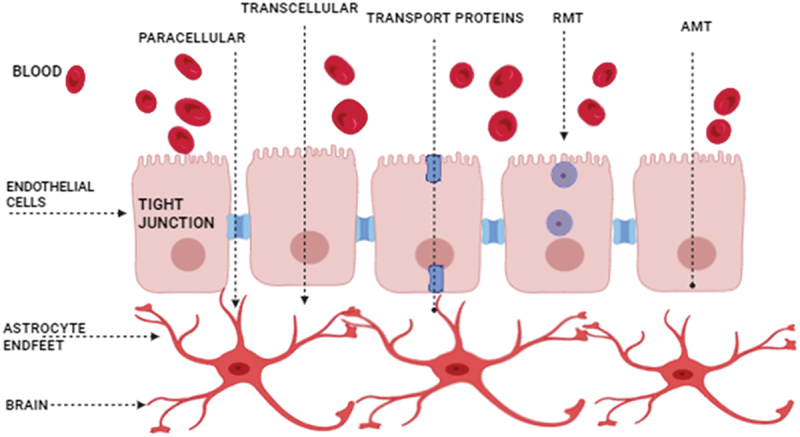
Transportation pathways via blood brain barrier.

Drugs are primarily absorbed via the BBB by means of two distinct processes, namely active and passive (Figure 4) [83,84]. Passive transport, also known as passive diffusion, refers to non-energetic transport channels that hydrophilic and lipophilic substances, respectively, utilize, such as transcellular and paracellular diffusion [85]. Paracellular diffusion: Local inflammation can disrupt the blood-brain barrier (BBB), weakening tight junctions and facilitating the transport of polar substances through endothelial cells [86]. Transcellular diffusion: The BBB allows for the passive diffusion of certain tiny, low-molecular-weight lipophilic compounds (400–600 Da) [73]. On the other hand, in active transport, materials are moved via energy-dependent processes that enable the passage of biological gradients like concentration or electrochemical ones [87,88]. Carrier-mediated transport: Specific protein carriers facilitate the transport of tiny molecules from the blood vessels towards the extracellular environment of the brain, including ions, amino acids, and glucose [89].

In particular, the glucose transporter 1 (GLUT-1) is responsible for carrying glucose [90,91], whereas the L system is unique for delivering amino acids, including valine, histidine, methionine, tyrosine, and phenylalanine, to the brain [92]. The alanine/serine/cysteine transporter (ASC) can then be used by neutral amino acids such as alanine, cysteine, and serine. Receptor-mediated transcytosis (RMT) refers to the process by which certain receptors on the inner side of endothelial cells in the brain parenchyma facilitate the movement of large and hydrophilic molecules, such as hormones and proteins. The ligand is readily absorbed after first binding to its particular receptor [93]. Adsorption-mediated transcytosis (AMT): This process, which requires the endocytosis of the charged compound’s vesicles, is most commonly used by peptides or proteins with a positive charge.

**Table:** 

Benefits of Nose to Brain Targeting	Reference
Targeted administration	[94,95]
Enhanced bioavailability	[96,97]
Lower Dose strength required	[52,98]
Ease of administration	[99,100]
Safety	[52,101,102]
Reduced systemic toxicity	[103–105]
Instant therapeutic action	[106–108]
Improved patient compliance	[109–111]
Non-invasive route	[103]
**Drawbacks of Nose to Brain Targeting**	
Clearance of the Mucociliary Tract	[45,112]
Complex nasal architecture/Complex anatomy of nasal cavity	[113,114]
Influence of nasal secretion on absorption	[115]
Low permeability for hydrophilic drugs	[116]
Short retention time	[117]

## 4. Challenges and limitations of nose-to-brain drug delivery

The challenges (Table 3) of nose-to-brain drug delivery were widely associated with the safety prospect and assessing the selective delivery efficiency.

**Table 2.** Various challenges and limitations of nose to brain drug delivery.

Challenges	Reference
If more than a sufficient amount of drug enters the nasal cavity, it can readily become saturated, which may result in the increase of drug level in the blood circulation and, as a result, cause side effects.	[101]
Regardless of the evidence that bioactive molecules use trigeminal and olfactory routes to reach the brain directly, several factors may hinder CNS drug absorption, resulting in a low concentration of the drug.	[118]
The nose-to-brain drug delivery has certain limitations, such as mucociliary clearance, a short residence period in the nasal cavity, drug degradation, and drug displacement by efflux mechanisms.	[107]
There are various potential limitations to the N2B delivery of drug-loaded nano-formulations. Despite the fact that intranasal drug administration can be beneficial still, the rate of absorption and permeability of organic products is still low. Some agents may have the potential to cause nasal mucosal toxicity; hence the toxicity evaluation is necessary prior to intranasal administration.	[119]
Delivery of peptide formulations from N2B requires appropriate formulation design to maximize the efficacy of drugs used in CNS disorders.	[120]
Clinical translation of these agents requires addressing issues such as nanomaterial potential toxicity, accurate dosing control, and sustained therapeutic efficacy.	[121]
Factors such as irritation to the mucosa and low residence time can lower the transmission of drugs as they increase the weight of the molecular drug, which restricts its entry only to a certain portion of the brain.	[97]
Certain drugs will be degraded by the enzymes present in the mucosa of nasal cavity resulting in reduce bioavailability via N2B drug delivery.	[122]
Intranasal formulations targeted to the brain can induce harmful effects as they require repeated administration of intranasal drugs, which may disturb the mucosal membrane as well as the olfactory nerves located there.	[123]
The surface area of the olfactory area present at the top of the nasal cavity is occupied by 5to7% of epithelial cells, and this restricts drug transmission, resulting in major drawbacks.	[109]
The mucus combined with ciliary movement in the nasal mucosa causes hindrance to drug as it restricts the drug motion towards CNS and its residence time in nasal cavity.	[124]
It is suitable only when a certain size of drug is to be delivered and not applicable for drugs that needs high dose to achieve their therapeutic efficacy.	[105]
Absorption occurring by epithelial cells of the olfactory region is limited for drug molecules having a weight above 100 Da because of poor permeability by the endothelial cells at the basement membrane.	[51]

## 5. Nanocarriers in nose-to-brain delivery

### 5.1. Polymeric nano-suspensions

Polymeric nano-suspensions are frequently used nanoformulations containing drugs, which are stabilised by lipid mixtures or non-ionic surfactants. Polymeric nano-suspensions provide several benefits, such as enhanced drug loading, simplified manufacturing, improved pharmacokinetics, and the possibility of surface modification [125]. The polymer matrix can protect the drug from degradation by enzymes in the nasal cavity, while also allowing for controlled, sustained release of the drug over time, further enhancing brain delivery. Nevertheless, the production of polymeric nanosuspensions is an intensive procedure, and they have not been selected as the primary option for treating chronic diseases due to their limited stability over time.

### 5.2. Polymeric nanoliposomes

Polymeric nanoliposomes are vesicles composed of a central aqueous compartment surrounded by a layer of lipids, which can be either single or multi-lamellar. The nanoliposomes’ structural design enhances stability, enables efficient drug encapsulation, and facilitates evasion of the reticuloendothelial system [126]. The liposomal bilayer facilitates the encapsulation of both hydrophilic and lipophilic drugs, protecting them from enzymatic degradation. The polymer coating improves mucosal adhesion, enhancing absorption. These nanoliposomes are taken up by nasal epithelial cells via endocytosis, allowing direct delivery of the drug to the brain, bypassing the blood-brain barrier. Although there is ongoing debate on the durability of nanoliposomes for treating neurological diseases, a study has demonstrated that curcumin nanoliposomes are particularly effective in combating amyloid aggregates [127].

### 5.3. Polymeric nanogels

Polymeric nanogels are formed by the process of emulsification and solvent evaporation using cross-linked amphiphilic or hydrophilic polymers [128]. The nanogel formulation is done by the combination of non-ionic and ionic polymers, resulting in the formation of cross-linked networks [129]. Polymeric nanogels are believed to offer superior protection to encapsulated pharmaceuticals during transportation compared to other types of nano formulations [49]. Their hydrogel structure swells in the presence of nasal fluids, facilitating drug release. The small size and high surface area of nanogels enhance mucosal adhesion and absorption. The drug is then permeated by nasal epithelial cells through endocytosis, enabling direct transport to the brain. Delivering DNA, siRNA, and oligonucleotides has been the primary use of polymeric nanogels, with encapsulation efficiencies varying from 40% to 60% [130]. Nanogels have demonstrated superior efficacy in delivering oligonucleotides to the brain compared to the spleen and liver [131].

### 5.4. Polymeric nano micelles

The hydrophobic core of polymeric nano micelles is encircled by a hydrophilic polymer block shell [132]. The shell of polymeric nano micelles provides stability and prevents cellular interactions, while the core can encapsulate approximately 30 % of hydrophobic drugs [133–135]. Polymeric nano micelles have shown their potential in delivering DNA both *in-vitro* and *in-vivo*. The hydrophobic core protects the drug from enzymatic degradation, while the shell facilitates drug release at the target site. Permeation occurs via endocytosis, allowing direct drug uptake by nasal epithelial cells and subsequent transport to the CNS. However, there is a lack of research on the use of nano micelles for delivering drugs to the central nervous system (CNS) [136]. PEGylated phospholipid nano micelles have been proven in vitro to inhibit amyloid-induced toxicity [137]. Nevertheless, polymeric nano micelles are unsuitable for encapsulating hydrophilic drugs. Furthermore, their shelf life has also decreased [138].

### 5.5. Nanocapsules and nanospheres

Nanocapsules are vesicular systems in which a thin, harmless polymer envelopes an oil-filled drug compartment, whereas nanospheres are solid-core polymeric matrices made using the micro-emulsion polymerization technique [139,140]. Both nanocapsules and nanospheres provide the benefits of improved drug stability, easy surface modification, and prevention of systemic breakdown. Nanocapsules and nanospheres can permeate the nasal mucosa through the tight junctions between epithelial cells, allowing the drug to pass directly into the bloodstream or reach the olfactory region. Nevertheless, they possess several drawbacks, including challenging purifying and storage processes, as well as unpredictable patterns of drug release [141]. Studies have shown that nanocapsules containing indomethacin may effectively shield hippocampal cultures from inflammation that occurs in laboratory settings [142].

### 5.6. Metal nanoparticles

Recent studies have focused on metal nanoparticles due to their potential applications in medical engineering and science [26]. Metal nanoparticles may be synthesized with diverse architectural and surface modifications, hence enhancing their potential utility in magnetic-based separation, precise delivery of genes and drugs, and particularly diagnostic imaging [143]. The surface properties of metal nanoparticles mainly the surface charge, can promote interactions with the epithelial cells, enhancing uptake and transport to the brain. Several existing and sophisticated imaging modalities, including SERS, CT, MRI, PET, and ultrasound, need the use of a contrast agent in order to operate well. The need for a contrast agent prompted the creation of nanoparticles made of gold, silver, and magnetite (Fe3O4) [144].

### 5.7. Silver nanoparticles

Human fibroblast cells, the lungs, and the skin have all shown cytotoxicity in response to silver nanoparticles (AgNPs) [145]. AgNPs have been found to pass the blood-brain barrier and accumulate in the brain after inhalation and ingestion [57]. Silver nanoparticles often possess surface charges (positive or negative) that facilitate interactions with the negatively charged nasal epithelial cells, improving cellular uptake and facilitating transcellular or paracellular transport. Patchin et al. [146] discovered that 20 nm AgNPs translocated rapidly into the olfactory bulb, but 110 nm silver particles transported slowly and ineffectively following a 6-hour treatment. An investigation found that there was a limited level of absorption of AgNPs (measured as total silver) into the bloodstream when administered intranasally. However, when AgNO3 was administered, there were much larger concentrations of silver in the blood. This indicates that the presence of silver in the blood was due to the release of silver ions from AgNPs. In vitro studies have demonstrated that AgNPs cause cytotoxicity in neurons [147]. However, the precise processes by which AgNPs cause neurodegeneration remain unknown, and further research is required. Contrary to the above information, the research discovered that AgNPs had remarkable anti-inflammatory characteristics, lowered the production of ROS, nitric oxide, and TNFα generated by LPS, and led to a reduction in microglial toxicity towards dopaminergic neurons [147]. As a result, additional research is required before making decisions on how to build future classes of safe AgNPs.

### 5.8. Gold nanoparticles

AuNPs, or gold nanoparticles, are commonly used as nanomaterials in drug delivery and imaging [56]. Research has shown that AuNPs exhibit limited specificity because they lack a selective component that can differentiate between targeted and non-targeted cells [148]. To deliver therapeutic chemicals to specific cells or tissues, researchers paired AuNPs with cell-targeting ligands. Due to their small size and surface properties, gold nanoparticles can efficiently permeate the nasal mucosa. They can cross epithelial cell layers through paracellular or transcellular routes, facilitating drug absorption into the bloodstream or directly into the brain. AuNPs have a large surface area, making them ideal for conjugating numerous peptides, proteins, aptamers, and antibodies. Nevertheless, these methods of conjugation are extremely sophisticated and system-specific, limiting their applicability across multiple systems. Furthermore, some compounds are unsuitable for use in therapeutic applications due to the potential toxicity associated with the use of surfactants, in particular, cetyltrimethylammonium bromide [149]. AuNPs for neuronal absorption can be obtained through two pathways: by crossing the blood-brain barrier (BBB) or by entering through the olfactory nerves. Researchers in a study effectively utilized a direct transport pathway from the nose to the brain to deliver theranostic polyfunctional gold-iron oxide nanoparticles loaded with miR-100 and antimiR-21 to glioblastomas (GBMs) in mice [150].

### 5.9. Magnetic nanoparticles

In reality, the nanoparticles with magnetic properties are called magnetic nanoparticles, or MNPs. As demonstrated by the BBB endothelium, they can create transient holes in cell membranes that improve drug distribution and targeting; this process is known as magnetoporation [58]. Magnetic nanoparticles (MNPs) have been applied in various biological fields, such as magnetic contrast agents, magnetic vectors, and magnetic hyperthermia and heating [151,152]. The application of an external magnetic field can guide and concentrate magnetic nanoparticles at a specific location, such as the olfactory region or brain, enhancing the precision of drug delivery and minimizing systemic distribution. Researchers created an oleic acid-coated Fe3O4 nanoparticle nanocarrier that absorbed short hairpin RNA in an effort to provide a viable PD therapy. It was demonstrated that these superparamagnetic nanoparticles inhibited α-synuclein’s harmful effects on the cells, decreased its expression, and prevented α-synuclein-induced cell death [153]. Another study found that when mesenchymal stem cells (MSCs) were administered intraperitoneal (IV) in a PD mouse model after being cultured with micrometre-sized iron particles, the MSCs successfully delivered the particles and enhanced neurobehavioral evaluation [154]. In a mouse model of Parkinson’s disease, dextran-coated nanoparticles of iron oxide have been shown to improve the therapeutic effectiveness of human MSCs by reducing dopaminergic neuron loss and promoting the differentiation of human MSCs into dopaminergic neurons [155].

### 5.10. Nanoemulsions

Oil-in-water (O/W) and water-in-oil (W/O) nanoemulsions are two types of NEs that are created when two immiscible liquids are dispersed and stabilized by adding the right surfactants and co-surfactants, with the right particle size being approximately 100 nm [156]. The majority of O/W nanoemulsions utilized for nose-to-brain drug administration are the various formulations [157]. The surfactants used in nanoemulsions stabilize the formulation and enhance the permeability of the nasal mucosa, promoting better absorption of both lipophilic and hydrophilic drugs. To create a risperidone nanoemulsion, the right amount of oil (Campul MCM) and tween 80 were mixed. Chitosan (0.50%, w/w) was added to the risperidone nanoemulsion to create a mucoadhesive formulation, which was then mixed for one hour. In vivo experiments were conducted on a specific animal, albino rats, to assess the drug distribution in the brain and blood. Two formulations of risperidone were administered intravenously (IV) as NEs, and risperidone solutions were regulated using technetium (99mTc) labelling.

When compared with the other formulations of risperidone NE and solutions, the conclusion section demonstrated that intranasal administration of the mucoadhesive chitosan-NE emerged more rapidly in the drug transportation into the CNS region [158]. In an additional study, rats were used in in vivo tests employing ergoloid mesylate in the form of ‘submicron emulsions’ and various formulations, such as intravenous (IV) and inhalation (IN) of API solution form. When compared to the administration of API solutions, the pharmacokinetic study’s findings on various formulations indicate that IN administration of ‘submicron emulsions’ yields superior results [159].

### 5.11. Niosomes

**Figure 5.**
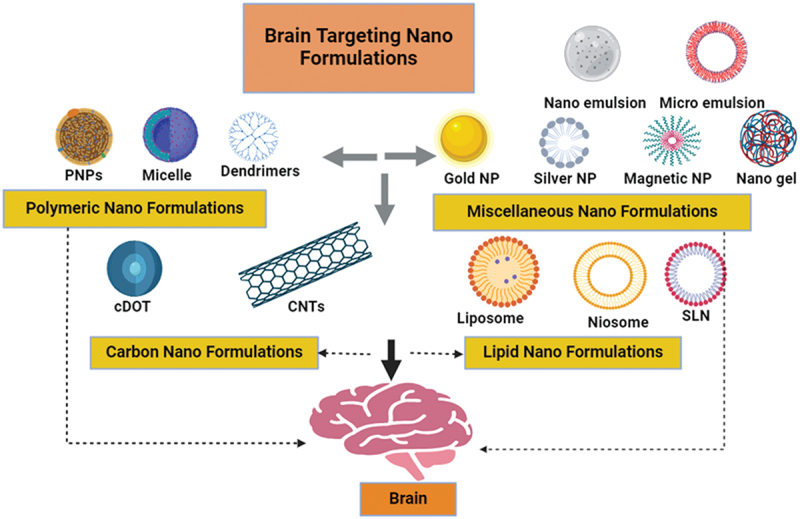
Nanoformulations for nose to brain drug delivery.

The primary components of niosomes, nanoscale vesicles, characterized by a persistent bilayer structure, consist of cholesterol and non-ionic surfactants. Highly biocompatible and biodegradable are niosomes [160]. Niosomes exhibit strong mucoadhesive properties, enhancing the residence time on the nasal mucosa. This prolonged adhesion increases the likelihood of drug absorption and targeting to the brain. They have low toxicity, a long shelf life, good chemical stability, and low manufacturing costs. Drug molecules can be delivered to the target site by niosomes in a sustained and controlled way. Niosomes can entrap lipophilic or hydrophilic medicines [161]. Niosomes have been shown to alter the metabolic stability and organ distribution of medicines. According to a study, the concentration of the atypical antipsychotic drug olanzapine in the brain increased thrice when the drug was present in surface-modified niosomes as opposed to the intranasal solution [162]. In order to treat Parkinson’s disease induced by subchronic MPTP therapy in C57BL-6J mice, a group of scientists developed a non-invasive technique of delivering the drug through the nose. This approach involves using niosomes, which are small vesicles wrapped with chitosan that contain pentamidine, or what we call pentasomes. According to the study, inPentasomes significantly improved parkinsonian motor dysfunctions By preventing the degeneration of dopaminergic neurons and decreasing the intensity of neuroinflammation in the nigrostriatal pathway; they were able to do this by inhibiting the activity of glial-derived S100B [163]. A related study described creating drug-free, pentamidine-loaded chitosan glutamate-coated niosomes for intranasal drug administration by the use of the thin-film hydration technique. In this study, particular attention was paid to investigating the interactions between mucin and niosomes loaded with pentamidine and those that were not. It was shown that pentamidine or other potential drugs might be efficiently administered to the brain through nasal inhalation using liposomal formulation [164]. Figure 5 displays a variety of nanoformulations used in brain targeting. Numerous formulations based on nanotechnology, along with their advantages and limitations, have been described in Table 3.

**Table 3.** Various advantages and limitations of nanoparticles for nose to brain delivery.

Nano formulation	Advantages	Limitations	References
Polymeric nanoparticles	High entrapment efficiency	Less biocompatible	[165,166]
Solid lipid nanoparticles	Increased bioavailability and controlled drug release pattern	Unpredicted particle growth.	[167,168]
Nano-emulsion and microemulsion	Non-toxic, increased bioavailability	A large number of surfactants are required for increased stabilization	[169–171]
Nanostructured lipid carriers	Targeted and controlled drug release, non-toxic	Issues with physical stability	[172]
Polymeric micelle	High drug loading and stability	Complicated polymer coating	[173]
Dendrimer-conjugate nanoparticles	Site-specific drug targeting	Toxicity	[174]
Lipid-polymer hybrid nanoparticles	Low frequency of administration, minimal adverse effects, prolonged release of the drug, and targeted delivery	Problems with stability and storage	[175]
Nanoparticles of Chitosan	improved absorption, stable, biodegradable, biocompatible, and non-toxic	The synthesis techniques are time-consuming and require the use of organic solvents in the preparation method.	[176]
PLGA nanoparticles	high loading capacity and increased penetration into tissues	Toxicity	[177]

## 6. Recent advancements in nanocarriers for nose-to-brain drug delivery

One of the most often prescribed anticonvulsant drugs for epilepsy, carbamazepine (CBZ), was included in a nanoemulgel developed by Samia et al. [178]. Combining nanoemulsion with a gel matrix, the nanoemulgel functions as a reservoir for drugs and prevents the degradation of enzymes. The adverse effect profile of CBZ, which can result in ophthalmic, pulmonary, renal, hepatic, hematologic, and cutaneous toxicity, is one of its main disadvantages. The researchers utilized an oil-in-water nanoemulgel technology containing xanthan gum, a mucoadhesive polymer, and oleic acid/labrasol in a 1:5 ratio to enhance the drug’s ability to target the brain specifically. This study has shown that the use of CBZ nanoemulgel intranasally (IN) might be a promising strategy for delivering the drug directly to brain in individuals with epilepsy [179]. The structural analogue of carbamazepine, oxcarbazepine (OX), has a lower rate of drug-drug interaction and a better pharmacokinetic profile. Nevertheless, due to its extensive distribution profile, which leads to reduced salt and bone density, it still has negative repercussions [180] in pentylenetetrazole-induced rats. For a period of 11 days, the subjects were given a daily amount of PLGA-OX IN as a pre-treatment. This resulted in a significant reduction in both the frequency and duration of symptoms, as evaluated by the Racine’s Convulsion Scale (RCS). Rats pretreated with PLGA-OX showed enhanced synthesis of brain markers, specifically neuro-filament and beta-tubulin, which was increased, whereas the expression of caspase activity was decreased [181].

Chitosan glutamate nanoparticles (CGNPs) were employed by Mittal et al. [119]. The purpose is to enclose rasagiline (RAS), which is a selective irreversible second-generation MAO-B inhibitor and a dopamine receptor agonist. Because of hepatic first-pass metabolism, RAS oral formulation has a short duration of action and limited absorption when taken orally, which may result in gastrointestinal side effects such as nausea and vomiting. Additionally, by reducing the drug’s crystallinity and enhancing its solubilizing activity, CGNP can speed up the drug’s rate of dissolution. The average DTE and DTP of RAS CGNPs were 325 ± 40% and 69.27 ± 2.1%, respectively, indicating more effective brain targeting with IN compared to IV [182]. It is shown that CGNP may be a viable delivery system for rasagiline to the brain, albeit additional research is needed to see how effective this method is.

Sunena et al. enhanced the capacity of galantamine, a distinct type of cholinesterase inhibitor, to adhere to mucus and be absorbed in the brain by encapsulating it in nanoparticles using thiolated chitosan (TC). Tripolyphosphate pentasodium (TPP) was electrostatically crosslinked with TC to create the nanoparticle. A pharmacodynamic investigation was carried out by assessing the ability of mice to recover from amnesia caused by scopolamine. In the Elevated Plus Maze, transfer latency was considerably reduced by galantamine-TC NP IN as compared to the scopolamine group and the scopolamine with blank NPs treated group. In Morris Water Maze models, galantamine-TC NP IN also considerably reduced acetylcholine esterase activity and The duration of time spent in the target quadrant in comparison to the galantamine administered intranasally. An advantageous result of administering galantamine-loaded targeted chitosan nanoparticles by nasal-to-brain delivery is the improvement of memory function and the suppression of acetylcholinesterase activity [183].

**Figure 6.**
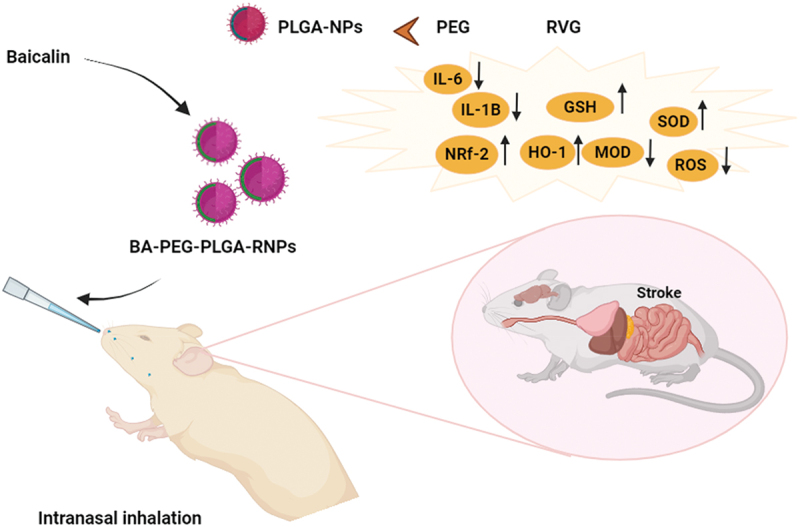
Brain targeting of baicalin polyethene glycol–polylactic acid–co–glycolic acid nanoparticles.

The topic of neuroprotection in cerebral ischemia (CI) is becoming more and more popular. However, because of the brain’s complex environment, effective therapeutic drug delivery to the brain continues to be a significant difficulty. Delivery via the nose to the brain is a potential strategy. Here, a recent study [184] synthesized RVG29 peptide-modified polyethene glycol – polylactic acid – co – glycolic acid nanoparticles (PEG – PLGA RNPs), which have physicochemical properties that lead to stable and sustained drug release and, consequently, improve the bioavailability of neuroprotective agents. This allowed us to optimize a nanocarrier formulation of neuroprotective agents that can be used for nose-to-brain delivery. In a rat model of CI, the brain-targeting capacity of PEG – PLGA RNPs delivered by nasal inhalation was confirmed as shown in Figure 6. The delivery agent for the neuroprotective drug was chosen to be baicalin (BA). Rats with ischemic brain injury showed remarkable improvements in their neurological dysfunction, reduced cerebral infarction area, and reduced nerve trauma and swelling following intranasal delivery of BA – PEG – PLGA RNPs.

Additionally, it was shown that BA’s neuroprotective benefits in a rat model of CI might be mediated by reducing oxidative stress and inhibiting inflammation. The ELISA findings demonstrated that the serum of rats suffering from cerebral ischemia had unusually high levels of TNF-α, IL-6, and IL-1β. Following treatment with BA-loaded nanoparticles, there was a considerable drop in the levels of TNF-α, IL-6, and IL-1β. Animals administered BA – PEG – PLGA RNPs by intranasal inhalation showed reduced levels of reactive oxygen species and malondialdehyde, increased glutathione and superoxide dismutase, and reduced oxidative stress. To sum up, rats can get BA from BA – PEG – PLGA RNPs and benefit from neuroprotective effects against CI [185].

To improve permeability and selectivity to the striatum, Tang et al. created co-modified nanoparticles (Lf-BNPs) with borneol and lactoferrin in addition to dopamine [186]. Bicyclic monoterpene borneol can improve the blood-brain barrier and nasal mucosa penetration of other therapeutic agents. The lactoferrin receptor is highly expressed at the apical surface of respiratory epithelial cells, capillaries, and neurons in the neurodegenerative brain [187]. According to a pharmacokinetic investigation, dopamine-Lf-BNPs had a brain AUC0–12 h that was significantly greater than the levels seen in Lf-NPs and dopamine NPs. Rats who were given apomorphine demonstrated contralateral rotation behaviour, and Lf-BNPs considerably reduced rotations compared to dopamine and control NPs. Furthermore, the striatum had the highest concentration of dopamine and its metabolites of all the groups, suggesting that it is possible to replenish dopamine levels effectively. This study demonstrated the tremendous potential of lactoferrin to improve delivery efficacy and borneol to promote nasal mucosa penetration.

Chitosan nanoparticles loaded with quetiapine were created by Shah et al. and then improved using the Box-Behnken design [188]. An SGA that reduces extrapyramidal symptoms is quetiapine fumarate (QF), a combination of serotonin type 2 (5-HT2) and dopamine type 2 (D2) antagonist. As a result of the first metabolism of substances by the liver, this drug has drawbacks such as a brief half-life and low oral bioavailability. Furthermore, quetiapine decreases the concentration of P-glycoprotein (P-gp) drug efflux pumps, which is a substrate for the drug. To overcome these problems, the authors employed chitosan nanoparticles, and they assessed the effectiveness and security of taking them orally. Overall, QF-NP’s intranasal delivery of QF demonstrated a good result; nonetheless, it may potentially be utilised for the treatment of schizophrenia.

Sharma et al. created polymer-based nanoparticles (NPs) that contain diazepam (Dzp). Dzp is a benzodiazepine with a quick onset and extended half-life that is frequently used as an antiepileptic, hypnotic, and anxiolytic [119]. Dzp’s lipophilicity allows it to be readily redistributed outside of the brain, which lowers the brain’s serum level of the drug. Dzp must be taken at multiple doses in order to sustain therapeutic efficacy. This study used PLGA as a carrier and the N2B delivery method to avoid systemic negative effects from multiple dosing and enable controlled release of Dzp. Within a time frame of up to eight hours after administration, tests using technetium-99 m-labeled (99mTc) Dzp showed that PLGA-Dzp IN had a greater ratio of drug concentration in relation to blood compared to Dzp solution administered by intravenous (IV) and intranasal (IN) routes (*p* < 0.05) [189]. This study proposes that PLGA-Dzp has the potential to be utilised to manage epilepsy.

**Figure 7.**
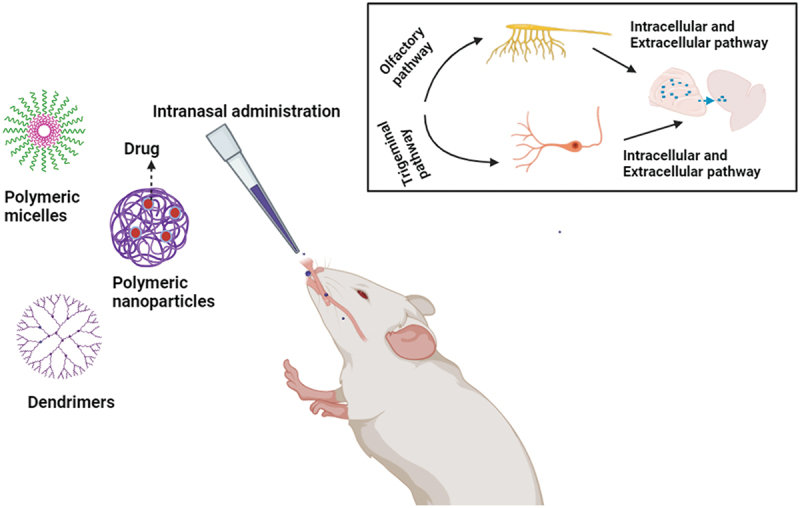
Polymeric structures in nose to brain delivery.

The efficacy of thymoquinone-loaded PLGA chitosan nanoparticles in the treatment of cerebral ischemia in mice was assessed by Xiao et al. [190]. A phytochemical called thymoquinone (TQ) was isolated from Nigella sativa seeds and can lessen damage caused by reactive oxygen species [156]. Nevertheless, TQ has a low solubility in aqueous solutions and is hydrophobic and light sensitive, which results in a low bioavailability. TQ-PLGA NPs IN exhibited superior AUC, Cmax, and half-life compared to TQ-PLGA NPs IV, according to a pharmacokinetic investigation conducted in the brain and plasma. It may be inferred that TQ PLGA NPs can be administered directly to the brain by intranasal delivery, as evidenced by the enhanced drug transport efficiency (DTE) and drug targeting percentage (DTP) of 524.17% and 80.47%, respectively [191]. Various intranasal delivery of polymeric structures have been depicted in Figure 7.

Pioglitazone (PIO), a peroxisome proliferator-activated receptor-gamma agonist, was encapsulated by Jojo et al. using NLC. Although it is frequently recommended to treat type II diabetes, a number of recent studies have suggested that it may also be used to treat Alzheimer’s disease by lowering oxidative stress [192]. Encapsulating PIO has the advantage of avoiding the blood-brain barrier during absorption, which helps reduce systemic adverse effects like peripheral oedema and bladder cancer. The brain/plasma ratio of PIO concentration in PIO NLC IN was substantially greater than that in PIO IN and IV groups (1.6 vs 0.84 vs 0.15, respectively), according to an in vivo biodistribution study. This suggests a lower risk of systemic toxicity [193]. Overall, the study demonstrated that PIO might be delivered to the brain; however, more research is required to determine whether PIO IN is beneficial in treating Alzheimer’s disease.

In order to improve the efficacy of Huperzine A-loaded PLGA nanoparticles for the treatment of AD, Meng et al. employed lactoferrin (Lf) and N-trimethyl chitosan (TMC) as ligands [194]. Although it is not authorized for treatment in AD, the cholinesterase inhibitor huperzine A can be taken as a dietary supplement to improve memory. Gastrointestinal adverse effects, including nausea, vomiting, constipation, and diarrhea, are among the main consequences of huperzine A. The study used the N2B delivery strategy in an effort to improve brain targeting while reducing the systemic negative effects of the medicine. The presence of Lf and TMC significantly enhanced the amount of drug buildup and mucoadhesion in the brain. Consequently, these nanoparticles may be employed for the efficient delivery of N2B.

Triolein was used by El-Zaafarany et al. to make Tween 80-coated emulsomes for triglycerides [195]. The primary distinction between an emulsome and a liposome is that the former has an aqueous inner core. In contrast, the latter has a solid lipid inner matrix that permits the loading of hydrophobic molecules in lipid bilayers. Due to its lipophilic properties, the OX emulsion IN exhibited a considerably greater brain AUC and shorter Tmax compared to OX PO and OX IV, as well as higher systemic absorption. OX emulsion IN’s DTP (62.3%) and DTE (265.7%) indicate that direct brain targeting via the olfactory pathway is more effective than using an indirect systemic channel from intranasal delivery.

To improve the effectiveness of drug transport to the brain, Md et al. developed chitosan (CS) nanoparticles loaded with bromocriptine for N2B delivery [196]. Levodopa is used in conjunction with bromocriptine (BRC), a dopamine receptor agonist derived from ergot. The primary drawback of this therapeutic agent is its quick hydroxylation in the liver, which results in reduced brain bioavailability. In order to get over this problem, BRC was given intravenously after being encapsulated in chitosan nanoparticles. When BRC-CS NP was supplied IN as opposed to IV, the relative bioavailability was 135.7 ± 14.05%, indicating that greater BRC may be obtained (*p* < 0.05). Additionally, the BRC-CS NP IN’s DTE and DTP values (633 ± 86.1% and 84.2 ± 1.9%, respectively) demonstrated that IN was preferred to IV [197].

An additional atypical antipsychotic was also considered for N2B administration. Polycaprolactone nanoparticles loaded with aripiprazole were employed by Sawant et al. for intranasal administration [198]. Third-generation atypical antipsychotic aripiprazole (APZ) binds highly to D2 and 5-HT2 receptors. Because APZ is metabolized extensively in the liver and is a P-gp substrate, dose escalation is necessary to maintain its therapeutic impact. This can result in a number of systemic adverse effects, including hypotension, hyperglycemia, and QTc prolongation. Since polycaprolactone has a low toxicity and good stability, it was utilized in the formulation of nanoparticles. Histopathological analysis revealed that while APZ-NP did not considerably harm the nasal epithelium, free APZ solution did cause considerable cilia destruction [119]. Though intranasal APZ-NP treatment generally increased the brain targeting effect compared to IV administration, the author’s conclusion was not supported by the DTE value (64.11 ± 4.68%).

## 7. Future perspectives of nanocarriers via nasal delivery

Nanocarriers have been showing encouraging results for the treatment of various neurological disorders through the intranasal route as it provides fast access to the brain without the need to pass through BBB [101]. Recent studies present the delivery of substances via the nasal route to the brain as a new approach to enhance brain targeting. Recent developments in the delivery of drugs via the nasal route combined with nanotechnology have opened a new scope for Parkinson’s disease treatment. However, more studies are needed to establish the efficacy of direct delivery from nose to brain [199]. Intranasal delivery coupled with nanocarriers seems to be a promising combination to give better clinical profiles, pharmacokinetics and pharmacodynamics for neurodegenerative disorders such as Parkinson’s disease, Alzheimer’s disease, schizophrenia and migraine. Nose-to-brain delivery can serve as a better alternative to the oral route with effective, targeted, noninvasive and safe delivery of neurotherapeutics [200]. Nose-to-brain delivery has proven to be the ultimate destination for the delivery of neurotherapeutics into the brain. However, to date, no confirmed mechanism for nose-to-brain delivery has been discovered. For the treatment of neurological disorders, nanocarriers are a reliable option for the intranasal route as they not only improve fundamental properties but also impart significant improvement in stabilization, solubilization and sustaining residence and release in the nasal environment [201].

Multiple aspects must be considered when manufacturing nanoparticles to develop a product that can reach patients. Safety of formulation, long-term chemical, physical and microbiological stability, and ease of scaling up are key parameters that should be considered. Synergistic interactions between academic, industrial and regulatory parties will be needed [107]. Significant anatomical and physiological differences exist in the nasal cavity of experimentally studied species and humans that should be considered while implying these experimental results to humans [202]. In nose-to-brain delivery, the restricted progress is due to the animal models, as they do not serve as the exact replica of physiological conditions and intricacies of human beings, and an interspecies variation exists. The differences between animal models and humans, such as variations in nasal anatomy, mucosal permeability, and metabolic processes, can significantly impact the translation of treatment outcomes, making it essential to refine preclinical models to better predict human responses and optimize therapeutic development. Optimal dosing regimen and administration conditions should be considered essential for the success of intranasal delivery of drugs. A strong research background for direct nose-to-brain delivery is needed in terms of the development of formulation and assessing their optimal delivery to the brain for bringing the nose-to-brain delivery to clinical premises [199]. In intranasal drug delivery systems, further development of nanoparticles promises to enhance the efficacy of the drugs and reduce resistance and adverse effects of the drugs by directly targeting the brain and increasing the availability of the drugs in the brain [203]. Work reported over the years suggests that optimizing the parameters of formulation, such as surface modification of liposomes, PEGylation, composition of phospholipids, use of different carriers, and use of edge activators, can enhance the bioavailability of the brain. One promising approach in optimizing formulations for nose-to-brain delivery is the use of chitosan-based nanoparticles, which have shown enhanced mucosal adhesion and improved drug absorption in preclinical trials. Additionally, the application of intranasal gel formulations, such as those incorporating muco-adhesive agents like carbopol, has demonstrated increased drug retention and prolonged release, leading to better therapeutic outcomes in clinical studies targeting neurological disorders. These technologies offer potential for more effective drug delivery to the brain.

Along with the optimization of formulation, the impact of the nasal environment on the liposomes should also be considered. For example, the drug release rate and stability of liposomes are affected by the changes in the tonicity in the nasal environment [204]. Multiple in vitro and in vivo investigations have been conducted to demonstrate the potential of nanocarriers in targeting drugs for the central nervous system (CNS), leading to promising clinical applications. With time advancements in technology, the synthesis of multifunctional and more advanced nanocarriers will be possible and can be used in treating several central nervous system diseases [14]. A future perspective for treating brain disorders is the utilization of nucleic acid as a therapeutic moiety. Nanoparticles can be used to deliver the nucleic acid to the target site. Several novel nanoscale carriers can locally deliver the drug to the brain thus increasing the targeting effect and control over the release of the therapeutic agents. Thus, in-depth knowledge of the nose-to-brain delivery system, the pharmacokinetic and pharmacodynamics studies of therapeutic agents used in these preparations, and the manufacturing of a device that is capable of efficiently delivering drugs to the olfactory site is important [119].

For an effective pharmaceutical action, the total drug dose reaching the targeting site is an important factor. In the delivery of drugs to the brain via the olfactory region, their transport and distribution in the brain play an important role. The study of effective dose and its relation to the drug biodistribution in the brain could be an emerging area for research [105]. However, the administration of nanocarriers through intranasal route is a potential topic for research due to several unsolved challenges. These include dealing with polar drugs having large molecular weight, mucociliary clearance, and degradation of drugs by enzymes in the lumen of the nasal cavity. With the advancement in nanotechnology for nose-to-brain delivery, it will be possible to improve their permeability, decrease peripheral toxicities, and increase the specificity of brain tissues [205].

## 8. Conclusion

The intranasal route is a recommended alternative drug delivery method over the oral and parenteral routes since it has numerous advantages over the other routes and can, therefore, overcome some of the restrictions. The greatest barrier to drug delivery to the brain and, thus, to the treatment of all CNS disorders, including tumor and neurodegenerative diseases, is the blood-brain barrier (BBB). Polymer nanoparticles (NPs) possess unique chemical and physical properties that enable them to function as systems capable of penetrating the blood-brain barrier (BBB) and directly delivering therapeutic substances to the brain.

Additionally, NPs can shield the drug from chemical and biological deterioration, boosting its bioavailability. Currently, researchers are utilizing several novel approaches, such as targeting ligands, nanoparticulate systems, and mucoadhesive formulations, to develop a potentially efficient intranasal drug delivery device that has little toxicity and unwanted effects. Currently, most of the investigations are in the preclinical or early clinical stages, relying solely on animal models to provide valid conclusions. It is predicted that the intranasal route may be a future approach for drug delivery to the brain, but very few studies have progressed to human clinical trials. Drugs seem to benefit from being included in nanocarrier systems such as liposomes, SLNs, nanoemulsions, microemulsions, nanosuspensions, polymeric nanoparticles, and dendrimers since they greatly reduce the risk of drug degradation and enhance brain targeting. Therefore, the use of drug-loaded nanocarriers for intranasal brain targeting has demonstrated significant promise for the treatment of several CNS diseases.

## Data Availability

All the data are contained in the manuscript.
