# Correction

**DOI:** 10.1080/15685551.2026.2606584

**Published:** 2025-12-25

**Article title**: A comprehensive insight of innovations and recent advancements in nanocarriers for nose-to-brain drug targeting

**Authors**: Naeem Mubarak, Muhammad Ahsan Waqar, Asad Majeed Khan, Zainab Asif, Aima Subia Alvi, Aqsa Arshad Virk and Sakeena Amir

**Journal**: *Designed Monomers And Polymers*

**Bibliometrics**: Volume 28, Number 01, pages 1-23

**DOI**: https://doi.org/10.1080/15685551.2025.2464132

We have identified that an incorrect page number was included in the catch line of this article. The corrected pagination has now been updated in the published version. This correction does not affect the description, interpretation, or original conclusions of the article. Taylor & Francis apologizes for any inconvenience caused.

